# Critical assessment of Islamic endowment funds (Waqf) literature: lesson for government and future directions

**DOI:** 10.1016/j.heliyon.2020.e05074

**Published:** 2020-10-13

**Authors:** Raditya Sukmana

**Affiliations:** Department of Islamic Economics, Faculty of Economics and Business, Universitas Airlangga, Jl Airlangga No. 4, Surabaya, 60286, Indonesia

**Keywords:** Endowment fund, Waqf, Awqaf, Islamic economics, Islamic social finance, Public economics, Economic development, Economic growth, Research and development, Religion

## Abstract

In this paper, the literature on Islamic endowment funds (awqaf) was surveyed. The data suggest that banking, accountability, law, and norms constitute most of the coverage in this body of literature. The *finance* topic, with 35% coverage, dominated the literature, whereas *history* (with 6% coverage) recorded the least interest. In this sample, the second most popular theme was related to economic development (25% coverage). Also discovered was that 59% of the papers employed a normative approach, and the balance adopted an empirical approach. A direct benefit of this study is that it provides input for government policy and directions for future researchers.

## 1 Introduction

This study presents an extensive survey of the available literature on Islamic endowment funds (Awqaf^1^) in reputable journals. The ultimate objective of this survey is to provide input for government policies to accommodate individuals' funds that support socioeconomic development. Indeed, as a government responsibility, poverty alleviation can be significantly supported by individuals' charitable efforts (Kaleem and Ahmed, 2009). A waqf is an endowment (donation) made by a Muslim under Islamic Law to a fund manager (mutawali/nazhir) who is responsible for generating profits that are subsequently used to support socioeconomic development. A waqf is similar to an endowment fund but is strongly encouraged in Islam as a contribution to society. A further objective is to guide researchers toward more quality research.

In doing so, this paper uncovers various findings from reputable journals and adopts strict criteria for selecting papers. High-quality papers ensure that a comprehensive and in-depth analysis is elaborated on in the papers. Recent papers are selected to ensure that the government has the latest waqf advanced model because old waqf papers that present old models might not be suitable in the current socioeconomic environment. A good journal is chosen to ensure that the government has studies that have high academic accountability. Moreover, researchers also receive benefits because this paper proposes further potential topics to be studied. After applying the rigorous criteria, only 63 papers remained.

The selected papers were then classified into five main topics, namely, Finance, Accountability, Law and Norms, Economic Development, and History. The major topic among the papers is Finance (35%), and History made the least contributions.

The sequence of this paper is as follows. The next section is about the methodology adopted in this study. Section 3 discusses all of the selected literature that has been classified into five topics and several of their subtopics. For the following section, because some of the collected papers discussed empirical waqf development in certain countries and because this development differed from one country to another, a brief discussion of waqf performance in selected countries is worthwhile. Next section is lessons learned for the government and proposed topics for future research and this paper ends with the section on conclusion.

## 2 Research methodology

This study has gone through several stages with the specific criteria to ensure that only high-quality papers were selected with the aim to provide the best recommendation for the government and further studies. When designing this research on the waqf literature review, this study gained benefits from several papers on the methodology for doing the research on the literature review. Snyder (2019), Hoque (2014), Aguinis and Glavas (2012), and Narayan and Phan (2019) are the main papers.

One of the concepts proposed by Snyder (2019) and Aguinis and Glavas (2012) is how papers were classified based on author, discipline, type, and contribution. This classification is vital to enabling readers to look into the state-of-the-art of certain topics and focus on how to extend them from specific topics. Furthermore, Snyder (2019) and, to some extent, Hoque (2014) stressed that the motivation for conducting a literature review is to ensure the quality of the selection process and to select only quality papers.

The motivation of this study, which provides recent high-quality research on awqaf, is twofold. First, in the past, awqaf (donated by individuals and non-government institutions) played a vital role in developing various sectors, such as health, education, and religion (Mohsin, 2013; Çizakça, 2015). Its role was important in lessening the burden of the government budget. Therefore, the government must understand the role of a waqf before designing socioeconomic development policies. Second, this paper aims to provide new ideas for further research based on the state of the art elaborated on in this study.

Moreover, two important benefits were realized from the study conducted by Aguinis and Glavas (2012). First, their paper elaborated on many issues, such as human resource management, marketing, and organizational theory within Corporate Social Responsibility (CSR). Second, their paper synthesized what was done in previous studies and provided a critical analysis of what is known (where we have been) and what is not known (where we need to go) regarding Corporate Social Responsibility.

Aguinis and Glavas (2012) and, to some extent, Hoque (2014) are used by this paper to elaborate on various aspects of a waqf. Additionally, this paper explains the current status on waqf research. Moreover, this paper is a significant extension of the work by Agunis and Glavas in the context of who benefits from a study of waqf research and how. In this case, this study finds that the government is the entity that benefits from this study (in response to who benefits), and this paper provides suggestions on the utilization of the waqf instrument regarding adoption as a government policy and alternative development tool (in response to how it benefits).

Moreover, filtering papers has the following four stages, as modified from Narayan and Phan (2019).

Identify journal database

First, this study selects the following seven commonly used journal databases: Science Direct, Emerald Insight, JSTOR, SpringerLink, Oxford Journal Academic, Cambridge Core, and Sage.

Use of keywords “Waqf,” “Awqaf,” and “Islamic Endowment Fund”

Second, these three keywords are used to search for papers.

Type of paper is Research Article

Third, the seven journal databases contain the following main types of papers: research paper, conceptual paper, general review, chapter item, review, case study, secondary article, and technical paper. This study focuses on the research article type of paper to ensure that the existing study is only based on research.

Publication year of 2010

Fourth, this study only considers papers published since 2010 because older waqf papers might offer models that are only suited for the period around which they were published. Given the current dynamic advancements in the economic, social, and technological environments, the current waqf model might be better than those of the past.

Papers that pass the previously described criteria are scrutinized on the basis of the following.

1. Scopus index: Selected paper is checked for whether the journal is indexed on Scopus using scimagojr.com.
2. Eliminate predator journal and/or publisher: In this case, the bell list predator journal (predatoryjournal.com) was used as a filter.
3. Waqf portion discussion: Lastly, the selected papers must have a dominant waqf discussion. In some papers, a waqf discussion ensues but as a small part of the larger theme of philanthropy. In this case, the paper is not included in this study.

These criteria were adopted for all seven databases. This study found 8,452 papers using the keyword “WAQF,” 2,564 papers using the word “AWQAF,” and 42,035 papers using the phrase “Islamic Endowment Fund^2^.” When “research article” was selected, the remaining 30,704 papers included 5,867 for “WAQF,” 1,521 for “AWQAF,” and 23,316 for “Islamic Endowment Fund.” When the publication year was limited, the 10,515 remaining papers included 1,105 for “WAQF,” 442 for “AWQAF,” and 8,968 for “Islamic Endowment Fund.” Then, the Scopus index, predator journal, and the waqf domination criteria were applied, which resulted in 63 papers. See Table 1.

**Table 1:** Process for filtering papers.

No.	Database	Keywords			Filter						
					Type “research article”			Publication Date 2010–2019			Scopus & full waqf
		“waqf”	“awqaf”	“islamic endowment fund”	“waqf”	“awqaf”	“islamic endowment fund”	“waqf”	“awqaf”	“islamic endowment fund”	
1	SpringerLink	940	435	2249	102	23	354	59	11	192	
2	Emeraldinsight	265	124	342	192	111	259	179	107	258	
3	Jstor	6421	1776	3617	5060	1241	2552	629	245	402	
4	Sciencedirect	182	51	585	138	42	398	71	25	247	
5	Oxford Journal Academic	363	99	1265	181	53	880	80	33	497	
6	Sage Journals	209	55	1877	150	42	1346	79	20	719	
7	Cambridge Core	72	24	32100	44	9	17527	8	1	6653	
Number of articles		8452	2564	42035	5867	1521	23316	1105	442	8968	63

## 3 Existing research

This section is divided into two parts. In the first part, this section presents studies classified into the five main topics of Finance, Economic Development, Law and Norms, Accountability, and History. Each topic is divided into several subtopics. For example, Finance has the largest number of papers and has the following five subtopics: Islamic Microfinance, Banking, Non-bank Finance, Financing Agriculture, Financing Education. Other subtopics are listed in Table 2. In the second part, toward the end of each topic, general lessons to be learned are discussed.

**Table 2:** Classification of Waqf research topic, subtopics, and papers.

Topic	Sub-topic	Papers
Finance	Islamic Microfinance	Haneef et al. (2014)
		Haneef et al. (2015)
		Ahmed and Salleh (2016)
		Thaker et al. (2016)
		Abdullah and Ismail (2017)
		Hamber and Haneef (2017)
		Zabri and Mohammed (2018)
	Banking	Ramli and Jalil (2014)
		Darus et al. (2017)
		Hamza (2017)
		Mohammad (2015)
	Non-bank Finance	Hasan and Sulaiman (2016)
		Rahman and Ahmad (2011)
		Zakaria et al. (2013)
		Abdullah and Saiti (2016)
		Thaker and Thaker (2015)
		Sulaiman et al. (2019)
	Financing Agriculture	Orbay (2012)
		Shafiai et al. (2015)
		Moh'd et al. (2017)
	Financing Education	Mahamood and Rahman (2015)
		Shamsudin et al. (2015)
Economic Development	Economic Development	Abdullah (2018)
		Hisham et al. (2013)
		Amuda et al. (2016)
		Noor and Yunus (2014)
		Shabbir (2018)
		Mohsin (2013)
		Shaikh et al. (2017)
		Suhaimi et al. (2014)
		Iman and Mohammad (2017)
		Sanusi and Shafiai (2015)
		Ambrose et al. (2018)
		Adeyemi et al. (2016)
		Sulaiman and Zakari (2019)
		Pitchay et al. (2018)
		Thaker (2018)
		Thaker et al. (2018)
Law and Norms	Law	Rahman and Amanullah (2017)
		Abbasi (2012)
		Beverley (2011)
		Akhtar (2012)
		Abdullah and Hudaib (2019)
		Siregar (2016)
	Norms	Pitchay et al. (2015)
		Khadijah et al. (2017)
		Noordin et al. (2017)
		Rizal and Amin (2017)
		Shukor et al. (2019)
		Jalil et al. (2019)
Accountability	Accountability	Masruki and Shafii (2013)
		Daud (2019)
		Nahar and Yaacob (2011)
		Yaacob and Nahar (2017)
		Ihsan and Septriani (2016)
		Ihsan et al. (2011)
		Ihsan et al. (2017)
		Yaacob et al. (2015)
		Azmi and Hanifa (2015)
History	History	Reichmuth (2010)
		Çizakça (2015)
		Khalfan and Ogura (2012)
		Igarashi (2019)

### 3.1 Finance

The Finance topic had the most papers, at 22 out of the 63 selected papers. These 22 papers were subdivided by subtopics, which are discussed next.

#### 3.1.1 Islamic Microfinance

Haneef et al. (2014) argued that the integration of awqaf and Islamic microfinance can alleviate poverty in Organization of Islamic Cooperation (OIC) countries. Haneef et al. (2015) proved that the Islamic Micro Finance (IWIM) integration model can alleviate poverty in Bangladesh. This research was conducted using a survey of 381 respondents, a literature review, and in-depth interviews with experts. Ahmed and Salleh (2016) also proposed a conceptual framework for inclusive Islamic financial planning (IFP) that integrates the issues of financial inclusion, financial planning, and financial literacy using the zakah and waqf concepts that ultimately aim to reduce poverty. Thaker et al. (2016) found that factors such as norms and attitudes have a positive impact on a microentrepreneurs' intentions to adopt the Integrated Cash Waqf Micro Enterprise Investment (ICWME-I) model in Malaysia. A cash waqf is suitable for Islamic microfinance institutions because such financing is very inexpensive for these entities (Abdullah and Ismail, 2017). Similarly, another example of a cash waqf supporting microenterprises is described for Singapore, which proposes waqf-based social micro-venture funds (Hamber and Haneef (2017)). The integration of cash waqf institutions (CWIs) and financial cooperatives (FCs) would provide affordable financing in the context of Malaysia (Zabri and Mohammed, 2018). This study offers a theoretical contribution regarding the ability of low- and middle-income earners to own houses.

#### 3.1.2 Banking

Ramli and Jalil (2014) analyzed the utilization of a waqf under “Wakaf Selangor Muamalat,” which was established jointly by Perbadanan Wakaf Selangor (PWS) and Bank Muamalat Malaysia Berhad (BMMB). This empirical study is the first on awqaf and recommends that other corporations cooperate with the Islamic banking industry, which adopted awqaf as a banking product. Darus et al. (2017) found that CSR within the Islamic banking industry prioritizes the internal as opposed to the external environment with respect to waqf involvement in contributing to the welfare of society. This finding is the result of the fact that CSR has limitations. Hamza (2017) argued that an efficient cash waqf structure is needed to allocate funds for investment and for considering the temporary impact of the risk level of liquidity, payments, investments, and the cost of bank funds. Mohammad (2015) found that a cash waqf can be utilized as a bank waqf operation.

#### 3.1.3 Non-bank finance

Hasan and Sulaiman (2016) argued that Islamic real estate investment trusts (I-REITs) can be adopted by waqf institutions as a mechanism to finance their waqf asset development. Rahman and Ahmad (2011) stated that the integration of awqaf and takaful products could support a wealth redistribution function among Muslims. Similarly, Zakaria et al. (2013) found that the integration of a waqf with a venture philanthropy model can reduce inequality. Abdullah and Saiti (2016) found that musharakah bonds for waqf property development have yet to completely fulfill the contract requirements and revenue generation necessary for shariah compliance. Thaker and Thaker (2015) revealed several issues related to regulations for managing waqf shares and, more specifically, taxation, payment modes, and dividend administration. Moreover, waqf unit trusts can be applied as an alternative strategy to ensure sustainable returns for beneficiaries (Sulaiman et al., 2019).

#### 3.1.4 Financing agriculture

Orbay (2012) suggested that awqaf can be utilized as an instrument to develop agriculture in Konya but that the natural environment and the social and political situations remain prime obstacles. Shafiai et al. (2015) opined that farmers should have access to finance mechanisms that may come from the integration of awqaf and Islamic banks dedicated to financing agriculture. Moh'd et al. (2017) reviewed the existing literature on financing models and made three proposals related to Zanzibar. First, waqf muzara'ah-based supply chains could be designed to solve the financing problem. Second, partnerships could help reduce the high interest rates and, finally, collateral could be included.

#### 3.1.5 Financing education

Mahamood and Rahman (2015) found that awqaf play an important role in financing education institutions (universities) in Malaysia and Turkey; thus, awqaf indirectly support the development of education. Shamsudin et al. (2015) proposed a conceptual cross-country waqf model that could finance higher education institutions.

#### 3.1.6 In general, what lessons can be learned?

Two important lessons on awqaf for financing can be learned.

First, the collaboration between awqaf and financial institutions may have a significant impact on development. Cash awqaf managed by financial institutions have zero costs of funds. The money being put as awqaf into a financial institution—other than a temporary cash waqf—does not need to be returned to the waqif (waqf giver), creating room for liquidity for banks and allowing them to conduct profit or nonprofit programs. The financial institution may finance a profitable project with the profits being channeled to social purposes. Alternatively, the financial institution can finance social programs at zero financing rates.

This collaboration can also reduce poverty (Haneef et al., 2014, 2015; Ahmed and Salleh, 2016), reduce the gap between the rich and the poor (Rahman and Ahmad, 2011; Zakaria et al., 2013), increase financial access (Hamber and Haneef, 2017; Moh'd et al., 2017; Abdullah and Ismail, 2017; Hamber and Haneef (2017); Zabri and Mohammed, 2018), create new banking products (Ramli and Jalil, 2014). and possibly establish waqf-based banks (Mohammad, 2015) that are distinct from other common banks. Moreover, awqaf can support the social and basic needs sectors, such as education and health (Mahamood and Rahman, 2015; Shamsudin et al., 2015) and agriculture (Shafiai et al., 2015; Orbay, 2012). Therefore, the government is expected to adopt this waqf instrument as important to socioeconomic development.

Second, evaluations, strategies, and innovations related to waqf-based financing need to be conducted for a number of reasons. Evaluation creates more efficient governance (Hamza, 2017) and improves rules and regulations for better waqf performance (Thaker and Thaker, 2015; Abdullah and Saiti, 2016). Meanwhile, strategies (Sulaiman et al., 2019) and innovations related to awqaf (Hasan and Sulaiman, 2016) can also improve the morality of the nazhir/mutawalli (Thaker et al., 2016). An example of a strategy is for banks to use CSR as a waqf. The previously described studies made a few recommendations. The government has to focus on the various types of waqf innovation through comprehensive and updated rules and regulations, particularly on the uses of CSR as a waqf. Moreover, the government should also address the negative impact of innovation because innovation may create disruptive economics through the existent of a larger number of unemployed.

### 3.2 Economic development

Abdullah (2018) found that 17 objectives of the Sustainable Development Goals (SDGs) promoted by the United Nations are in line with the long-term objectives of shariah. Stakeholders can undertake developments on the basis of awqaf for the SDGs framework. Hisham et al. (2013) opined that istibdal in Malaysia—if well-managed—can support sustainable economic development. Amuda et al. (2016) suggested that cash awqaf can also positively affect human development and, thus, is an important aspect of economic development. Noor and Yunus (2014) suggested that Build, Operate and Transfer (BOT) schemes offer a shariah option that is compatible with a waqf.

Shabbir (2018) used the analytical hierarchy process (AHP) and divided waqf land into the four sectors of agriculture, commercial, residential, and religious—important factors for socioeconomic development and that may create many job opportunities. Mohsin (2013) stated that cash awqaf (managed by non-governmental organizations) should be channeled not only into religious sectors but also other sectors, such as education, health, commercial activities, and infrastructure, for greater benefit. Waqf institutions differ from other social organizations that primarily rely on donations from others (not guaranteeing sustainability) (Shaikh et al., 2017).

Empirically, Suhaimi et al. (2014) showed that endowment fund schemes play an important role for the Muslim community in Penang Malaysia. Iman and Mohammad (2017) argued that waqf-based entrepreneurship is also an alternative for promoting the welfare of society. Sanusi and Shafiai (2015) conducted a study on two waqf institutions in Malaysia and proved that awqaf contribute to the religious, economic, and social sectors. Moreover, awqaf have been implemented by the Malaysian government to finance public goods (Ambrose et al., 2018). This study is based on semi-structured interviews of academicians who specialize in awqaf and relevant government and private institutions, as well as available studies.

However, according to Adeyemi et al. (2016), the challenges are the awareness that cash awqaf are still low because of social and cultural problems and the lack of their promotion in Malaysia. Similarly, the sustainability of the waqf institution is an issue, given that only one out of seven waqf institutions in Malaysia are financially sustainable, which is based on the few components adopted in the study (Sulaiman and Zakari, 2019). Moreover, the existence of many idle parcels of waqf land encouraged Pitchay et al. (2018) to propose a cooperative waqf model that creates a relationship between a waqif (waqf donor) and a waqf institution that is aimed to make idle waqf land productive. This model puts the status of the waqif as a waqf project member, which has the special right to receive benefits from commercial projects developed on idle waqf land. A crowdfunding waqf model (CWM) can also be adopted to support the waqf institution in optimizing idle waqf land in Malaysia (Thaker, 2018). Implementing this CWM requires an IT system as an enabler to integrate all aspects of the operational waqf (Thaker et al., 2018).

#### 3.2.1 In general, what lessons can be learned?

Previous discussions stress the collaboration of awqaf and financial institutions. To a large extent, this section discusses awqaf in relation to economic development.

First, a waqf is an instrument that ensures the sustainability of economic development, which is in line with the SDGs (Abdullah, 2018) introduced in 2015 by the United Nations and general economic development (Hisham et al., 2013; Suhaimi et al., 2014). The utilization of a waqf is very flexible relative to that of zakah. For zakah, beneficiaries are already fixed, as is taught in the Islamic religion, but the beneficiaries of a waqf can be anyone (more general). Zakah and awqaf are complementary in supporting economic development (Mohsin, 2013; Sanusi and Shafiai, 2015; Amuda et al., 2016; Shaikh et al., 2017; Ambrose et al., 2018) and awqaf in the form of land, building, and cash certainly create more jobs (Shabbir, 2018).

Second, awareness of the waqf should be another important point to consider and requires a proper socialization program (Noor and Yunus, 2014; Iman and Mohammad, 2017) to ensure the sustainability of the waqf itself (Adeyemi et al., 2016; Sulaiman and Zakari, 2019). Empirically, abundant idle waqf land exists, and various attempts have been made regarding making such land productive (Pitchay et al., 2018; Thaker, 2018; Thaker et al., 2018). Therefore, universities need to create innovative waqf socialization programs that can have massive effects through various available alternatives, such as social media and newspapers.

### 3.3 Law and Norms

The third classification is Law and Norms, which each has six papers.

#### 3.3.1 Law

Rahman and Amanullah (2017) investigated the implementation of a temporary cash waqf in several states in Malaysia and found that improvements in procedures and legal issues, among others, are still required.

Abbasi (2012) focused on hanafi jurisprudence and found that family waqf law needs to be harmonized with the laws of inheritance and gifts. Beverley (2011) explained the criticisms of Muslim law activists of colonialism and the procedures in the law on waqf. Akhtar (2012) argued that the waqf status provision can function as a way to reduce the tax burden in England—as that of charity. Abdullah and Hudaib (2019) showed that awqaf and trusts have similar functions, but also have differences in their religious doctrines. If awqaf are based on the shariah (divinity), trusts are ruled by humans and may change from time to time.

The motivation of awqaf and trusts are also different. An individual's motivation to donate to a waqf is for the reward hereafter, whereas for a trust, the motivation might only be for worldly gain. Another important point is the governance of endowment management and not as much the general rules and regulations of a foundation (Siregar, 2016). The impact of this point is on the foundation's attempt regarding tax issuance. Paying less taxes is normally the objective, given fewer regulations on the governance of an endowment.

#### 3.3.2 Norms

Pitchay et al. (2015) revealed that attitudes and subject norms are important factors in relation to their cash waqf contributions through salary deductions. Khadijah et al. (2017) showed that religiosity, altruism, personal satisfaction, and commitment are among the personal characteristics that explain waqf preferences. The performance measurement system of Noordin et al. (2017) is an important step toward promoting good governance and morality within waqf institutions. Rizal and Amin (2017) indicated a significant relationship among perceived benevolence (Ihsan), Islamic egalitarianism, and Islamic religiosity and cash waqf contributions. Shukor et al. (2019) identified that an important factor in determining an individual's intention to give a cash waqf is trust in the waqf institution.

The trust of a waqif is influenced by integrity and institutional reputation. This reputation can be developed through the waqf institution's openness, honesty, and transparency with the public. Moreover, a waqf institution is to maintain the confidentiality of waqif data. Any data leakage ruins the integrity of the waqf institution.

Jalil et al. (2019) indicated that the roles of trust, communication, and payment method do not have a direct relationship with waqif commitment. Instead, these roles only act as moderating variables. Meanwhile, other factors, such as information disclosure, basic information, financial and non-financial information, information on future activities, and information on governance, have a direct relationship with waqif commitment.

#### 3.3.3 In general, what lessons can be learned?

Two important lessons can be learned from the aspect of laws and norms. First, laws on awqaf should be in line with the laws that are regulated by each country. Aspects of the laws of awqaf should be improved and ensure harmony with other related laws (Rahman and Amanullah, 2017; Abbasi, 2012; Beverley, 2011) to have a positive impact on society (Akhtar, 2012).

The second lesson is regarding the religiosity and morality aspect of waqf governance. These aspects can support waqf contributions and preferences (Pitchay et al., 2015; Noordin et al., 2017; Khadijah et al., 2017; Rizal and Amin, 2017; Shukor et al., 2019; Jalil et al., 2019).

Lessons learned from the aspect of laws and norms are related to the issue of the establishment of standard-setting for waqf governance. To date, the only standard-setting body related to Islamic financial institutions is the Islamic Finance Service Board (IFSB). However, the IFSB focuses on producing standards for Islamic banking, Islamic capital markets, and Islamic insurance—more of a business focus. However, a standard-setting body that focuses on social orientation is yet to be established.

### 3.4 Accountability

Nine papers were found on the issue of accountability. A waqf study on the conceptual framework was conducted by Masruki and Shafii (2013), who stressed the importance of accounting and accountability in waqf administration and management. For the future, this study recommends the introduction of accounting standards that are suitable for waqf institutions. Daud (2019) showed the importance of Islamic governance waqf reporting on the issue of transparency. This study recommends a few strategies for improving the governance of Islamic institutions.

A larger number of studies empirically investigated waqf accountability issues. Nahar and Yaacob (2011) and Yaacob and Nahar (2017) found that waqf accountability in management, accounting practices, and reporting in the context of Malaysia requires improvement to ensure the production of high-quality reports. Ihsan and Septriani (2016) proposed accountability in current practice given past examples. Ihsan et al. (2011) conducted an empirical study of accountability practice on two waqf institutions in Indonesia and showed that ABC^3^ (a waqf institution) is more efficient, transparent, and accountable relative to that of XYZ (another waqf institution) because ABC is managed by people who are committed and professional. Ihsan et al. (2017) empirically evaluated the accountability practices of Dompet Dhuafa and found that Dompet Dhuafa has been successfully integrating accountability and a commitment to preserve the organization's values, especially to the Mutawalli. A similar empirical study was conducted by Yaacob et al. (2015) in the case of Waqf-S Management. However, a waqf institution does not always follow AAOIFI standard No. 33 (SS 33) on awqaf. This study proposes two important aspects of disclosure/reporting on a shariah basis for a waqf institution (Azmi and Hanifa, 2015). These two aspects reflect the importance of waqf funds as dictated by waqif and modifications to waqf financial reporting.

#### 3.4.1 In general, what lessons can be learned?

Lessons from these studies are related to the importance of specifically complying with waqf accounting reports. Accountability in reporting is vital for the waqf institution as an aspect of transparency (Nahar and Yaacob, 2011; Yaacob and Nahar, 2017; Masruki and Shafii, 2013; Daud, 2019). Transparency is not a contemporary issue (Ihsan et al., 2011, 2017; Yaacob et al., 2015; Azmi and Hanifa, 2015) but, rather, has been accomplished in the past (Ihsan and Septriani, 2016).

Transparency and accountability within a waqf institution as part of a nonprofit organization need to improve because their roles are important in socioeconomic development. Therefore, for the waqf institution to attract stakeholders, transparency and accountability have to be at the forefront of the performance indicator, and misinformation from the stakeholder's perspective must be reduced.

### 3.5 History

Reichmuth (2010) describes the 1920 waqf document collection held at the national archives in Tashkent, Uzbekistan (CGA). The structure of these documents is compared with that of existing waqf documents. Çizakça (2015) argued that waqf research from a historical perspective until today continued to develop. Furthermore, the use of Islamic waqf in the west and China is also elaborated on. Khalfan and Ogura (2012) explored the management aspects of how to conserve a historical building in Zanzibar using Islamic waqf and found that good management systems can protect it from external threats. Igarashi (2019) analyzed a waqf by Qijmās al-Isḥāqī, a leader in Egypt during the Mamluk and Suriah dynasties, and showed that waqf systems have multidimensions and complex functions.

#### 3.5.1 In general, what lessons can be learned?

The fact that, in the past, awqaf were central to socioeconomic development is a good lesson for current governments when managing socioeconomic development. This important role can be learned from the waqf document heritage (Reichmuth, 2010; Igarashi, 2019). Moreover, awqaf were important to maintaining the historical heritage (Khalfan and Ogura, 2012). Given limited studies in a good journal on successful past awqaf, academicians need to engage in further research on this issue. Such research is important as input for current governments to optimize awqaf for socioeconomic development.

## 4 Waqf performance in selected countries

Awqaf play a vital role in the history of Islamic civilization. The Prophet Muhammad was the initiator of the waqf practice in many forms, such as a waqf of a piece of land that could be utilized for pious purposes and booty from war being used as a waqf for equipment for Muslim soldiers. After the prophethood, the waqf practice was continued by the companion. During that time, all awqaf were classified by type and size, starting from house, land, equipment for war, and water source (Muljawan et al., 2016). All of these awqaf were used for socioeconomic development in that area.

Awqaf developed rapidly during the Islamic reign. As more countries embraced Islam, the growth of awqaf followed because of the large amount of war booty used for general social purposes—not only for the poor and needy but also for knowledge development. A judge was appointed as the manager (nazhir) to monitor all waqf assets and ensure that the corpus was maintained and developed. Taubah Bin Namr was the judge and nazhirin of Egypt for the first time during the administration of Hisyam bin Abd al Malik. Moreover, during the Abbasiyah period, a judge was no longer appointed as nazhir. Since then, growth in development accelerated. A famous school, Al-Mustanshiriyah, is funded by a waqf (Muljawan et al., 2016). After the Abbasiyah Period, a waqf was developed by the next dynasty, al-Fatimiyah, al-Batiniyah, al-Ayubiyah, al-Mamalik, al-Bahriyah in Egypt and Syam, and al-Utsmaniyah in Turkey and some Arabic countries.

In today's contemporary world, the waqf management approach adopted by a country might differ from that of another country. Some of the collected papers on awqaf described a few country cases. Hence, observing the characteristic of a few countries (Malaysia, Singapore, Indonesia, and Turkey) when dealing with awqaf is worthwhile.

### 4.1 Malaysia

Among countries used as examples for the waqf study, Malaysia attracted the strongest interest from researchers, indicating that Malaysia, with an approximate 50% Muslim population, is very serious in developing this instrument. Awqaf as part of charity have been in Malaysia for centuries. Awqaf contribute significantly to the development of religious sectors, such as mosques, cemeteries, and orphanages (Rahman and Ahmad, 2011). The waqf practice in Malaysia follows the rules and regulations within the states. This practice has been specified in the Malaysian Constitution, which explains that any issues related to Islamic Laws (including awqaf) are regulated by the enactment of the state. Each state may have different approaches to dealing with awqaf, but the basic characteristics of awqaf remain the same in all jurisdictions within states.

As a federation country, Malaysia has 13 states and three federal territories, namely, Kuala Lumpur and Putrajaya in west Malaysia and Labuan in east Malaysia. Each state has a State Religious Council or Majelis Agama Islam Negeri (MAIN), which takes care of issues related to religion, including zakat and waqf (Ramli and Jalil, 2014). MAIN plays the role of sole trustee (nazhir) of awqaf. In other words, people who want to register their land as a waqf must approach MAIN. No other institution is allowed to receive awqaf other than MAIN—also implying that awqaf are centralized within their respective states.

To ensure good governance of awqaf in all states and given that significant waqf asset potential exists that can be optimized for socioeconomic development in Malaysia, the federal government has established two agencies—Perbadanan Waqf, Zakat and Haji (JAWHAR) and Yayasan Waqf Malaysia (YWM). These federal agencies aim to create effective and efficient waqf management throughout the country to ensure that the Malaysian people receive benefits. In doing so, these agencies have created a waqf development plan as part of the 9^th^ and 10^th^ Malaysian Plan (Ramli and Jalil, 2014). Moreover, the government gave these agencies full authority to develop waqf assets in cooperation with all of the MAIN throughout Malaysia. The planning included, for example, developing a boarding house for the orphanage in the state of Kedah (for social) and a waqf-based hotel in the state of Negeri Sembilan for commercial purposes.

#### 4.1.1 Singapore

Singapore is a small country with a minority Muslim population. The population is dominantly Chinese, followed by Melayu (majority Muslim) and Indian. Although the Muslims do not dominate the population, waqf performance as part of charity can be an important lesson learned by others. All awqaf in Singapore are managed by Majelis Ugama Islam Singapore (MUIS), which was enacted in the Administration of Muslim Law Act (AMLA) in 1968. During that time, individual nazhir existed, and many did not report to MUIS (Karim, 2011). Hence, MUIS was unable to obtain the exact number of awqaf in Singapore and could not support and make productive the idle waqf land held by incapable individual nazhir.

Therefore, in 1995, this AMLA was amended and started to optimize the idle waqf land. For example, waqf land on Duku Road was developed into a housing complex with a rental fee of SGD 68 in the early years. In 2005, the rental fee was SGD 36,000 per year. The second example is the extension of the mosque on Changi Road, which was developed into 20 apartment units. This building is funded by Musharakah bonds in the amount of SGD 60 million. The strategic location of the building enables the operational mosque expenditures to be covered by the profits from this property business (Muljawan et al., 2016).

The cash waqf in Singapore is also important to learn. According to Islamic law, awqaf are not obligatory. However, in Singapore, they are treated as obligatory. The amount of money that is automatically deducted from salaries depends on the designed bracket. For Muslim workers with a salary of less than SGD 1,000 per month, SGD 1 is deducted. For another bracket, such as workers with a salary of more than SGD 4,000 per month, SGD 7 is deducted. From this cash waqf, SGD 6 million was collected in 2005 (Karim, 2011).

#### 4.1.2 Turkey

Turkey has a rich experience with both productive and nonproductive awqaf. These instruments are vital to support sectors that are important to humans, such as health, education, and others. The golden period of awqaf was during the Ottoman period, during which awqaf numbered in the thousands (Heper et al., 2018). Moreover, awqaf contributed capital injections into several cities during the Ottoman period (Çizakça, 2000). During this period, 46% of the total waqf was in the form of a cash waqf, and the remaining was a property waqf (Muljawan et al., 2016). Furthermore, according to Kuran (2001), two-thirds of the fertile land in Turkey was identified as a waqf until 1923. This fact indicates that a waqf was the backbone for socioeconomic development during the Ottoman period, which also indicates that society's general awareness, in addition to that of professional waqf managers (nazhir), was high.

As time went by, awqaf gained greater appreciation. The Republic of Turkey replaced the Ottoman Empire and established the Waqf General Directorate, which aimed to follow up on all jobs that existed during the Ottoman period. This institution is directly responsible to the prime minister (Heper et al., 2018).

Various regulations on awqaf were accommodated under Turkey civil law. Çizakça (2000) explained the four waqf regulations. First, a waqf must have an executive board (chapter 77), and the waqf General Director supervises (chapter 78). Third, a waqf must be audited a minimum of once every two years. Fourth, the Waqf General Director as a supervisor and an auditor has the right to obtain 5% from waqf net income.

Furthermore, Çizakça (2000) identified that, in 1987, awqaf managed by a waqf general director were comprised of 4,400 mosques, 5,348 shops, 2,254 apartments, and other types, such as dormitories and business centers. Other than that, this institution has cooperated with other parties that also manage awqaf, particularly for investments, such as Ayvalik and Aydem Olive Oil Corporation, Tasdelen Healthy Water Corporation, Auqaf Guraba Hospital, Taksim Hotel (Sheraton), Turkish Is Bank, Aydir Textile Industry, Black Sea Copper Industry, Construction and Export/Import Corporation, and danTurkish Auqaf Bank (Muljawan et al., 2016).

Besides awqaf in the business sector, the waqf General Director also managed awqaf in the form of facilitating health, free dormitories for students, and others. Moreover, the socialization of awqaf to the general public continued to increase individuals' awareness to donate more awqaf.

#### 4.1.3 Indonesia

The Waqf Act, issued in 2004, was the first act on awqaf and marked a significant milestone in the history of awqaf in Indonesia. The act accommodates various issues related to awqaf, including types (including a cash waqf), duration (temporary or perpetual), and description of nazhir, waqif, and others.

According to the Waqf Act, the type of asset to be treated as a waqf can be in any form as long as the corpus remains intact. Land is commonly used for a waqf, cash awqaf are also accommodated in the Waqf Act to pave the way for people who do not have land but have cash to donate as a waqf. Moreover, the Waqf Act creates the opportunity for a waqif who wants to temporarily donate assets into a waqf, except for land. Therefore, for a waqif who wants to donate land into a waqf, it must be perpetual. However, if the waqif wants to donate money (cash), a duration is applied, which isimportant to explain in the Waqf Act to accommodate a waqif who wants to donate a cash waqf, either perpetually or temporarily.

According to the Waqf Act, a nazhir can be in the form of an individual or an institution. However, given the complexity of the issues once a waqf is developed, that the nazhir be in the form of an institution is encouraged. An institution is preferred because it has divisions that take care of various issues, such as legal, finance, and marketing, and can be established to cater to the specific aspects of waqf issues.

According to the Act, Badan Waqf Indonesia (BWI) is a waqf regulator and may simultaneously receive a waqf (as a nazhir) from the public. Moreover, a nazhir may be a private institution provided that is licensed from BWI. Therefore, anyone who wants to donate assets/property has two options regardless of whether he goes to BWI or private nazhir institutions.

BWI has branches in Indonesian provinces. Within the provinces, BWI has offices in almost every city. Therefore, each BWI in the cities is expected to develop waqf assets surrounding its areas because they know the empirical situation better than a BWI in a province or even headquarters.

The data from BWI show that Indonesia has more than 420 Hm^2^ of waqf land. Much of this land is in the form of mosques, graveyards, and schools, among others. The cash waqf potential reached Rp 188 trillion or USD 12.5 billion (BWI, 2020). However, cash waqf realization is Rp 185 billion or USD 12.3 billion, which was collected from 52 registered nazhir (BWI, 2020). This finding suggests that socialization of awqaf, including cash awqaf, must be more effective.

## 5 Analysis

This study modifies and extends the research by Narayan and Phan (2019) by making two contributions. This study provides input for government policies and identifies loopholes that need to be investigated in further studies. In this section, the discussion is separated into lessons for government policies and directions for further research. Summaries of the survey findings and the lessons learned are provided in Tables 1, 2, 3, 4, and 5.

**Table 3:** Summary of the Finance topic.

Sub-theme	Papers	Findings	What can be learned	Lessons for Governments	Further study for research
Islamic Microfinance	Haneef et al. (2014)	Integration of awqaf and Islamic microfinance can alleviate poverty in Organization of Islamic Co-operation (OIC) countries.	• Waqf integration plays an important role as financing to solve various types of problems and, at the same, time create innovation. • Evaluation, innovation, and waqf strategy need to be conducted for a few important things.	• Governments shall create a policy to introduce a cash waqf for microfinance institutions as new instruments for microenterprises that previously had a high cost of funds. • Governments shall create a policy to treat some or all of the CSR of Islamic Banking as waqf (endowed fund). • To create a policy to facilitate affordable houses for the poor, the government can design I-REITs with waqf instruments. • Governments shall give incentives for waqifs (waqf donors) who donate a cash waqf for the basic needs of the people, such as food (agriculture), education (by establishing schools), and health (by establishing hospitals).	• Comprehensive analysis of microenterprises that received cash waqf financing is needed. • Proposed model of cash awqaf and I-REITs is studied and validated by a focus group discussion by relevant parties, such as waqf regulator, Islamic capital regulator, and others. Islamic contracts underlying cash waqf financing of basic needs sectors, such as agriculture and education, is a good topic for future research studies.
	Hamber and Haneef (2017)	The integration model with Islamic microfinance (IWIM) can alleviate poverty in Bangladesh.			
	Ahmed and Salleh (2016)	Integration of financial inclusion and financial planning, financial literacy with the concept of zakah and waqf in a model called inclusive Islamic financial planning (IFP).			
	Thaker et al. (2016)	Norm and attitude have positive impacts on microentrepreneurs' intentions to adopt the Integrated Cash Waqf Micro Enterprise Investment (ICWME-I) model in Malaysia.			
	Abdullah and Ismail (2017)	A cash waqf is suitable for Islamic microfinance institutions because the financing given to them is very inexpensive.			
	Hamber and Haneef (2017)	The case of Singapore, which proposes waqf-based social microventure funds.			
	Zabri and Mohammed (2018)	Integration Cash Waqf Institutions (CWIs) and Financial Cooperatives (FCs) can facilitate affordable houses in Malaysia.			
Banking	Ramli and Jalil (2014)	Recommend that other corporations cooperate with the Islamic banking industry on waqf, for instance, by Perbadanan Wakaf Selangor (PWS) and Bank Muamalat Malaysia Berhad (BMMB).			
	Darus et al. (2017)	Corporate social responsibility (CSR) within Islamic Banking prioritizes more the internal environment, as opposed to the external, when it comes to waqf involvement in the welfare of society because CSR has limitations.			
	Hamza (2017)	An efficient cash waqf structure is needed for allocating funds for investment and considering the temporary impact on the risk level of liquidity, payments, investments, and bank costs of funds.			
	Mohammad (2015)	A cash waqf can be utilized as a waqf bank operation.			
Non-bank Finance	Hasan and Sulaiman (2016)	Islamic Real Estate Investment Trusts (I-REITs) can be adopted by waqf institutions as a mechanism to finance their waqf asset development.			
	Rahman and Ahmad (2011)	Integration of waqf and takaful products could support the Muslim wealth redistribution function.			
	Zakaria et al. (2013)	A philanthropic approach can be used in the context of waqf to reduce inequalities.			
	Abdullah and Saiti (2016)	Operation of musharakah bonds in waqf property development is yet to completely fulfill the contract requirements and revenue for shariah compliance.			
	Thaker and Thaker (2015)	Issues in managing waqf shares exist with the regulation, particularly taxation, mode of payment and dividend administration.			
	Sulaiman et al. (2019)	Unit Trust Waqf can be used as waqf strategic investments for sustainable development.			
Financing Agriculture	Orbay (2012)	Waqf can be one of the instruments utilized in developing agriculture in Konya, but the natural environment and the social and political situation have negatively influenced agriculture development.			
	Shafiai et al. (2015)	There should be mechanisms for the farmer to access finance, which may come from the integration of waqf and Islamic banks dedicated to financing agriculture.			
	Moh'd et al. (2017)	Three things were proposed for the case of Zanzibar. First, a waqf muzara'ah-based supply chain to solve the financing problem; second, a partnership to reduce the high interest rate; and, lastly, the existence of collateral.			
Financing Education	Mahamood and Rahman (2015)	Waqf plays an important role in financing education institutions, in this case a university; thus, indirectly waqf supports education development.			
	Shamsudin et al. (2015)	Propose a cross-country waqf model that could finance higher education institutions.

**Table 4:** Summary of the Economic Development topic.

Sub-theme	Papers	Findings	What can be learned	Lessons for Governments	Further study for research
Economic Development	Abdullah (2018)	Seventeen objectives of the SDGs are in line with the long-term objective within shariah. Stakeholders can make developments on the basis of waqf on the SDGs framework.	• Waqf is an instrument to ensure sustainable development • It requires effort to increase the awareness of waqf and optimizing the waqf asset.	• Governments could treat waqf as a mainstream instrument for economic development (SDGs) to lessen the burdens on the government budget. • Government should create policies on utilizing idle strategic waqf land and establish commercial properties using the Build Operate Transfer method. The profits generated to be used for social purposes. • Introduce the waqf concept at elementary and high schools.	• Among the elements of the SDGs are the health and education sectors. Further study of this issue could evaluate success stories on the use of waqf on health and education. • Another important study on the level of awareness of the people could be undertaken. This is important for marketing purposes.
	Hisham et al. (2013)	The argument is that istibdal in Malaysia, if it is managed well, can support sustainable economic development.			
	Amuda et al. (2016)	Cash waqf collection can have a positive impact on human development.			
	Noor and Yunus (2014)	Build, Operate and Transfer (BOT) scheme offers shariah option, which suits waqf development.			
	Shabbir (2018)	Categorize waqf land and prioritize the four sectors, namely, agriculture, commercial, residential, and religious.			
	Mohsin (2013)	A cash waqf is to be channeled not only into the religious sector but also other sectors that many people need, such as education, health, commercial activities, infrastructure.			
	Shaikh et al. (2017)	The advantage of waqf is its flexibility on the uses for the beneficiaries.			
	Suhaimi et al. (2014)	The endowment fund scheme plays an important role in economic development in the Muslim community in Penang.			
	Iman and Mohammad (2017)	Waqf-based entrepreneurship can be an alternative for the welfare of society.			
	Sanusi and Shafiai (2015)	Waqf is proven to contribute to religious, economic, and social sectors by conducting a study on the two waqf institutions in Malaysia.			
	Adeyemi et al. (2016)	Awareness of a cash waqf is still low due to social and cultural problems, and less promotion in Malaysia.			
	Sulaiman and Zakari (2019)	Only one out of seven waqf institutions ensures financial sustainability.			
	Pitchay et al. (2018)	Cooperative-waqf model reflects an increase in participation in developing idle waqf land.			
	Thaker (2018)	Crowdfunding-waqf model (CWM) developing idle waqf land using information technology.			
	Thaker et al. (2018)

**Table 5:** Summary of the Law and Norms topic.

Sub-theme	Papers	Findings	What can be learned	Lessons for Governments	Further study for research
Law	Rahman and Amanullah (2017)	The implementation of a temporary cash waqf in several states in Malaysia; found that improvements such as procedures, legal, and others are needed.	• Waqf law has to be in line with the law of individual countries. • The importance of religiosity and morality in waqf governance.	• To evaluate waqf institution performance, there should be a performance measurement. • In any policy, the government should make the environment, wherever possible, as religious as possible, to attract people to donate waqf.	• An important study yet to be conducted is to propose a model of a performance measurement system that has been validated by experts on waqf. • Another important study yet to be conducted is on waqf and taxation and its impact on the economy.
	Abbasi (2012)	Study focuses on hanafifiqh, and one of the findings is that family waqf law needs to be harmonized with laws on inheritance and gifts.			
	Beverley (2011)	Explained the criticism by Muslim law activists of colonialism on the procedure within the law on waqf.			
	Akhtar (2012)	The waqf status provision can function as a way to reduce the tax burden in England.			
	Abdullah and Hudaib (2019)	Waqf and trust have similarities in their functions but differ in term of the issue of doctrine (only available in waqf).			
	Siregar (2016)	Requires a specific legal structure for endowment management and not for the general law for a foundation.			
Norms	Pitchay et al. (2015)	Muslim labor has more influence relative to the subjective norm over their contribution to a cash waqf via salary deductions.			
	Khadijah et al. (2017)	Religiosity, altruism, personal satisfaction, and commitment are among the characteristics that explain waqf preferences.			
	Noordin et al. (2017)	Performance measurement systems are important to promoting good governance and morality within waqf institutions.			
	Rizal and Amin (2017)	Compassionate perception and egalitarian can influence religiosity for Muslims when donating waqf.			
	Shukor et al. (2019)	The trust of the waqif is an important factor in donating a cash waqf.			
	Jalil et al. (2019)	Basic information, financial information, and governance information have a direct relationship with waqif commitment.			

### 5.1 Finance

#### 5.1.1 Lessons for government

Governments need to create policies to introduce a new cash waqf for microfinance institutions to lessen their burden of microenterprises that previously had to bear the high costs of bank funds. By adopting a cash waqf, the financing rate can be significantly reduced.

CSR is commonly designed to benefit the company in the long run. Products by microenterprises are expected to be raw materials for companies that engage in CSR. However, this CSR could be integrated with waqf instruments through government regulations, whereby microenterprises only repay the principal borrowed.

In many countries, poor people cannot afford to buy houses. An Islamic real estate investment trust and waqf concept could be designed to lower and make affordable housing prices; hence, enabling the government to help poor people own a house.

The waqf donated (by individuals) to the health and education sectors certainly lessens the burden on government budgets. Therefore, governments may consider creating policies to attract more awqaf by providing incentives, such as tax rebates.

#### 5.1.2 Further study

To the best of the author's knowledge, no single study has investigated a comparison between new entrepreneurs who receive cash waqf financing and those who receive normal financing and, in particular, how quickly the businesses become successful. This topic is important to pursue in future research.

Regarding houses for the poor, a proposed model for the integration of cash awqaf and I-REITs should be studied and validated through focus group discussions attended by relevant important parties. Before the model can be applied, a policy study is needed to obtain a comprehensive and in-depth analysis of it.

An Islamic contract underlying cash waqf financing for basic needs sectors, such as agriculture and education, are other good topics for future research studies.

### 5.2 Economic development

#### 5.2.1 Lessons for government

Regarding the recent issues related to SDGs, almost all 17 indicators of the SDGs can be contributed to by awqaf. Therefore, awqaf should be treated as mainstream financial instruments for SDGs. Governments should create policies that ask nazhir/mutawalli (fund managers) to obligatorily utilize all of the idle waqf land that they manage with proper supervision from experts for the purpose of schools, medical clinics, and others through, for example, Build Operating Transfers (BOTs).

Furthermore, the government should introduce the waqf concept at an early stage of a child's education, such as elementary school and high school. The curriculum should be designed in such a way that it is not merely a concept or theory but that students are also given chances to practice accordingly.

#### 5.2.2 Further study

Successful cash awqaf for socioeconomic development (such as health and education) are many. Therefore, studies on the key success factors in managing a waqf should be continuously conducted in various places.

Compared with other sectors of Islamic economics, awqaf have been left behind despite their potential for significant impacts on socioeconomic development. Therefore, increasing people's level of awareness of the possibilities of awqaf is important and a key factor for the marketing of waqf institutions.

### 5.3 Law and Norms

#### 5.3.1 Lessons for government

Awqaf received by a nazhir/mutawalli (fund manager) must be utilized for the maximum benefit of the people; therefore, waqf institutions carry a significant responsibility to the public. To achieve this objective, the government needs to conduct regular assessments of waqf institutions. Also important is having a yardstick for such assessments. Therefore, a performance measurement for the assessments is required as part of government policy.

Previous studies indicated that religiosity is important to the people who donate a waqf. Therefore, wherever possible, government policy should ensure that people have a continuous inclination toward religion. With that in mind, more waqf donations are expected.

#### 5.3.2 Further study

Researchers have built various models to measure the performance of awqaf. Although finding a perfect model is difficult, at least a proposed and comprehensive model that has been discussed by experts and validated by relevant parties should be a sufficient yardstick for relevant parties.

To the best of the author's knowledge, currently, no studies deal with awqaf and the associated taxation issues or their impacts on the economy. Studying this topic is important.

### 5.4 Accountability

#### 5.4.1 Lessons for government

That waqf accounting standards are binding to all waqf institutions is essential to evaluate the performance of waqf institutions and, in particular, their financial reporting (see Table 6).

**Table 6:** Summary of the Accountability topic.

Sub-theme	Papers	Findings	What can be learned	Lessons for Governments	Further study for research
Accountability	Masruki and Shafii (2013)	• The importance of the use of accounting on waqf administration and management. For the future, it is expected to have accounting standards suitable for waqf institutions.	• The importance of designing waqf accounting standardization.	• Government may issue waqf accounting standards for all waqf institutions. • Government needs to conduct meetings with other governments to establish a standard-setting body for waqf governance, such as BASEL for banking governance.	• A comparison of the accounting treatment of waqf institutions needs to be conducted. • Research on accountability of waqf and a comparison with similar instruments such as endowment funds are needed.
	Daud (2019)	• It requires Islamic governance to ensure waqf report transparencies.			
	Nahar and Yaacob (2011) Yaacob and Nahar (2017)	• Waqf accountability in management, accounting practices, and reporting in the context of Malaysia, although it exists, needs improvement to ensure high-quality reports.			
	Ihsan and Septriani (2016)	• Propose accountability in the current practice, given the examples of the past.			
	Ihsan et al. (2011)	• Waqf institution ABC is more efficient and accountable compared with WYZ. Commitment and professionalism are key.			
	Ihsan et al. (2017)	• Dompet Dhuafa has been successfully integrating accountability as well as a commitment to preserving the organization's value.			
	Yaacob et al. (2015)	• Waqf-S is a very successful waqf manager. Administration and management are carried out in the most effective manner with comprehensive rules, guidelines, and procedures.			
	Azmi and Hanifa (2015)	• Waqf institution does not following AAOFI rule no. 33 on awqaf.			

Moreover, currently, no standard-setting body exists for waqf institutions such as BASEL for the banking industry. Therefore, governments in some countries need to initiate discussions that aim to establish such a body. In the future, the possibility exists that cash awqaf are donated by waqif in Country A to the nazhir/mutawalli (fund manager) in Country B, and are utilized for a profit-generating project in Country C for beneficiaries in Country D. For such cases, the governance of waqf institutions in all four countries have to be agreed on. Hereby, a standard-setting body for waqf governance gains importance.

To gain a different perspective, for the government to establish an association of international waqf regulators is better, for which each country can share their waqf operations for the benefit of others. For example, Malaysia and Indonesia have different approaches to conducting a waqf. In Malaysia, the fund manager (nazhir) is the government, which is not the case of Indonesia. In Indonesia, private institutions can be the nazhir that manages awqaf. Therefore, sharing is important to gain different perspectives.

#### 5.4.2 Further study

Because no guidelines on waqf accounting treatment currently exist, waqf institutions may receive different treatment regarding the waqf received from the waqif. Hence, comparing different treatments of awqaf and their effects on financial performance would be interesting.

To some extent, awqaf are similar to endowment funds. Both are donations, but awqaf differ because they are part of Islamic teaching that encourages all Muslims to participate. Given this difference, comparing the accountability of the reporting of both institutions (awqaf and endowment funds) would be interesting.

### 5.5 History

#### 5.5.1 Lessons for government

Awqaf played an important role in the past; however, because of colonialism, many waqf assets have been destroyed. To gain people's awareness of the impact, for the government to focus on waqf history is important. This focus can be in the form of waqf documents, archives, pictures, stories, and others, and would be an important resource for further studies (see Table 7).

**Table 7:** Summary of the History topic.

Sub-theme	Papers	Findings	What can be learned	Lessons for Governments	Further study for research
History	Reichmuth (2010)	• Describes in general the 1920 waqf document collection in the national archives of Uzbekistan in Tashkent (CGA). It is compared with the existing and available waqf information.	• The vital role of waqf in the past can be the role model for current waqf development.	• Waqf has played an important role in the past; however, many waqf assets were destroyed because of colonialism. To gain people's awareness of its impact, the government must establish a historical focus on awqaf, which can be in the form of waqf documents, archives, pictures, stories, and others, and would be an important resource for further studies.	• To the best of the author's knowledge, no single study was undertaken during the golden age of Islam on the issue of waqf and government revenue. This is a potentially important resource for the government as an alternative to taking out loans.
	Çizakça (2015)	• Waqf research on historical perspective until today exists nowadays and is developed.			
	Khalfan and Ogura (2012)	• Analyzed the management aspect of how to conserve the historical building in Zanzibar with Islamic waqf and found that good management systems can protect it from external threats.			
	Igarashi (2019)	• Waqf system has multidimensions and complex functions after analyzing waqf documents by Qijmās al-Isḥāqī.			

#### 5.5.2 Further study

To the best of the author's knowledge, no single study exists that was undertaken during the golden age of Islam on the issues of waqf and government revenues. However, a waqf is a potentially important financial resource for the government as an alternative to taking out overseas loans.

## 6 Conclusion

To conclude, this study is a response to the recent interest in waqf development, which is signaled by an increasing number of research articles on awqaf. To the best of the author's knowledge, 15 years ago, only a few papers—possibly none—that discussed awqaf were indexed by Scopus. This study collected 63 papers on awqaf that were filtered according to our strict criteria. This study intentionally accommodates only papers published from 2010 onwards, and the loopholes and recommend areas for future research on awqaf are described.

In the final selection of papers, the topic of finance is the most discussed, appearing in approximately 35% of the papers, whereas economic development reached 25%. Studies on the aspects of law and norms and accountability achieved rates of 19% and 14%, respectively. Issues on the history of awqaf were found to be the least discussed, at 6%.

Second, regarding the type of research study, 37 papers were found on the normative approach, and the remaining 26 papers were focused on the empirical implementation of awqaf. This study is expected to provide future researchers with many avenues for their studies.

## Declarations

### Author contribution statement

All authors listed have significantly contributed to the development and the writing of this article.

### Funding statement

This work was supported by 10.13039/501100008463Lembaga Penelitian dan Inovasi (LPI) Universitas Airlangga, Indonesia.

### Competing interest statement

The authors declare no conflict of interest.

### Additional information

No additional information is available for this paper.

## Footnotes

1 Awqaf is the plural form of waqf.

2 An attempt was made to use the keywords “Islamic endowment” but the result was the same; that is, no single papers met the criteria.

3 Not a real name.
